# Influence of Physiological Variables and Comorbidities on Plasma Aβ40, Aβ42, and p-tau181 Levels in Cognitively Unimpaired Individuals

**DOI:** 10.3390/ijms25031481

**Published:** 2024-01-25

**Authors:** Francisco Martínez-Dubarbie, Armando Guerra-Ruiz, Sara López-García, Juan Irure-Ventura, Carmen Lage, Marta Fernández-Matarrubia, Ana Pozueta-Cantudo, María García-Martínez, Andrea Corrales-Pardo, María Bravo, Juan Martín-Arroyo, Jon Infante, Marcos López-Hoyos, María Teresa García-Unzueta, Pascual Sánchez-Juan, Eloy Rodríguez-Rodríguez

**Affiliations:** 1 Neurology Service, Marqués de Valdecilla University Hospital, 39008 Santander, Spaincarmen.lage@gbhi.org (C.L.); magarcia.mart@gmail.com (M.G.-M.); martin.arroyo.juan@gmail.com (J.M.-A.);; 2 Institute for Research Marqués de Valdecilla (IDIVAL), 39011 Santander, Spain; 3 Biochemistry and Clinical Analysis Department, Marqués de Valdecilla University Hospital, 39008 Santander, Spain; 4 Immunology Department, Marqués de Valdecilla University Hospital, 39008 Santander, Spain; 5 Atlantic Fellow for Equity in Brain Health, Global Brain Health Institute, University of California, San Francisco, CA 94143, USA; 6 Health Sciences Department, Universidad Europea del Atlántico, 39011 Santander, Spain; 7 Network Center for Biomedical Research in Neurodegenerative Diseases (CIBERNED), 28220 Madrid, Spain; 8 Medicine and Psychiatry Department, University of Cantabria, 39011 Santander, Spain; 9 Molecular Biology Department, University of Cantabria, 39011 Santander, Spain; 10 CIEN Foundation, Queen Sofia Foundation Alzheimer Center, 28220 Madrid, Spain

**Keywords:** Alzheimer’s disease, plasma biomarkers, presymptomatic stages, comorbidities, amyloid, tau protein

## Abstract

Plasma biomarkers for Alzheimer’s disease (AD) are a promising tool that may help in early diagnosis. However, their levels may be influenced by physiological parameters and comorbidities that should be considered before they can be used at the population level. For this purpose, we assessed the influences of different comorbidities on AD plasma markers in 208 cognitively unimpaired subjects. We analyzed both plasma and cerebrospinal fluid levels of Aβ40, Aβ42, and p-tau181 using the fully automated Lumipulse platform. The relationships between the different plasma markers and physiological variables were studied using linear regression models. The mean differences in plasma markers according to comorbidity groups were also studied. The glomerular filtration rate showed an influence on plasma Aβ40 and Aβ42 levels but not on the Aβ42/Aβ40 ratio. The amyloid ratio was significantly lower in diabetic and hypertensive subjects, and the mean p-tau181 levels were higher in hypertensive subjects. The glomerular filtration rate may have an inverse relationship on plasma Aβ40 and Aβ42 levels but not on the amyloid ratio, suggesting that the latter is a more stable marker to use in the general population. Cardiovascular risk factors might have a long-term effect on the amyloid ratio and plasma levels of p-tau181.

## 1. Introduction

Alzheimer’s disease (AD) is the most prevalent cause of dementia [1] and is characterized by the deposition of extracellular amyloid-β (Aβ) plaques and intraneuronal neurofibrillary tangles of phosphorylated tau (p-tau) in the brain [2]. These protein changes are reflected in cerebrospinal fluid (CSF), and its analysis constitutes one of the main diagnostic tools [3]. However, a lumbar puncture (LP) is an invasive technique that cannot be used in a population-based setting. In recent years, it has become technically possible to accurately measure various forms of Aβ (Aβ40, Aβ42) and p-tau (p-tau181, p-tau217, p-tau231) in plasma, opening up the possibility of a non-invasive and cost-effective diagnosis [4,5]. This is especially relevant now that the first disease-modifying drugs are being approved, and screening tests will be required to use them.

AD markers in plasma can be now measured, but their significance at different stages of the disease and the factors that influence their levels are still not entirely clear. On the one hand, there are laboratory factors that influence them in different ways. P-tau181 presents a significant inter-subject variability (≈18%), while the Aβ42/Aβ40 ratio presents the lowest intra- and inter-subject variability (≈3%) [6]. Moreover, AD has been related in the literature to several pathologies, especially cardiovascular risk factors, and previous studies have evaluated the influence of different physiological variables and comorbidities on plasma levels of Aβ and p-tau [7,8,9].

Although the importance of blood pressure, dyslipidemia (DLP), and diabetes mellitus (DM) has been studied, the most frequently reported factor is renal function. Thus, individuals with chronic renal failure have been shown to have higher levels of plasma Aβ and p-tau than those of healthy individuals [10,11]. However, these studies have been mainly performed by measuring plasma biomarkers with SIMOA (single-molecule array) or ELISA (enzyme-linked immunosorbent assay) technologies, and there is very little information about automatic immunoassays, such as Lumipulse, a promising and widely available technique that allows the measurement of AD markers in a fully automated way.

It is essential to take all these factors into account before we can use plasma markers in a clinical setting so that we can interpret amyloid and tau levels according to the comorbidities and physiological variables of each subject. With our study, we want to explore the importance of different serum parameters and biometric variables on plasma levels of AD markers in cognitively unimpaired (CU) participants. This information on the preclinical stages of AD could help to implement plasma biomarkers as a screening tool. For a better understanding and interpretation of our results, we also performed the same analysis on CSF biomarkers.

## 2. Results

### 2.1. Sample Description

In our sample of 208 subjects, 65.4% were women, with a median age of 64 years (IQR 60–69). A total of 30.3% were carriers of at least one ε4 allele of the *ApoE* gene. The biochemical and biometric variables and both plasma and CSF marker levels are summarized in Table 1.

### 2.2. Influence of Comorbidity Status on Plasma Markers

**Figure 1:**
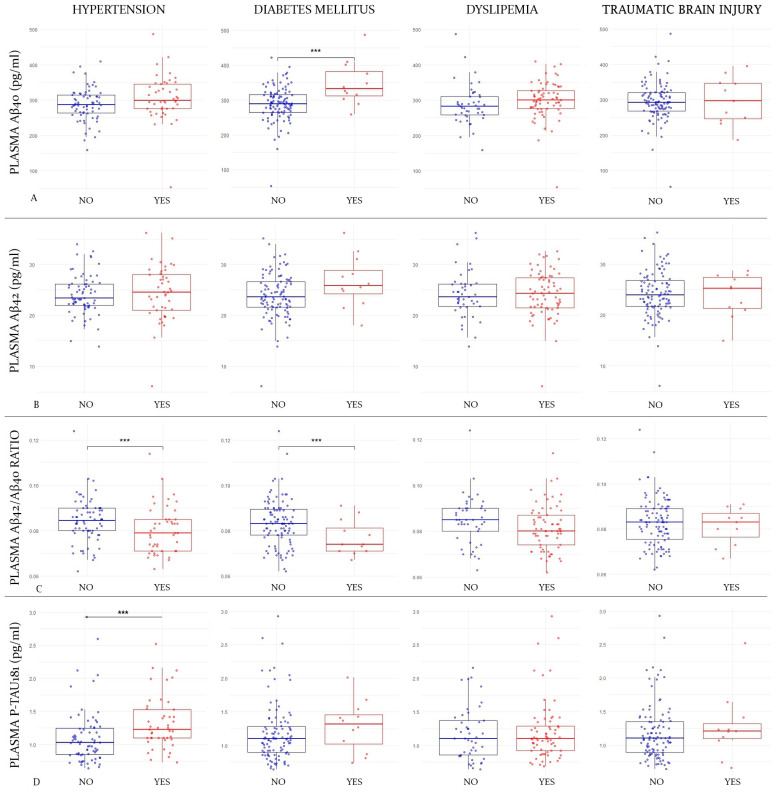
Differences in mean plasma marker values according to comorbidities.

Subjects with DM showed higher plasma Aβ40 values than those of non-diabetic subjects (347.8 vs. 288.6 pg/mL; *p*-value = 0.0002; Cohen’s d = 1.15). This difference was also significant in females (336.3 vs. 289.7 pg/mL; *p*-value = 0.005) but not in males (382.4 vs. 286.4 pg/mL; *p*-value = 0.28). However, there was no difference in Aβ40 values between subjects with and without HT (287.5 vs. 306.1 pg/mL; *p*-value = 0.07), DLP (287.3 vs. 299.7 pg/mL; *p*-value = 0.22), or TBI (294.6 vs. 296.6 pg/mL; *p*-value = 0.9) (Figure 1A).

Figure 1 shows box-and-whisker plots of plasma markers by comorbidity group. The X-axis represents the different groups according to the comorbidity status (hypertension status in the first column, diabetes status in the second one, dyslipidemia in the third one, and traumatic brain injury in the fourth). The Y-axis corresponds to plasma marker concentrations expressed in pg/mL (where applicable). The boxes show the interquartile range (the upper boundary is Q3, and the lower boundary is Q1). The line inside the box corresponds to the median of the sample, and the whiskers represent the maximum (upper) and minimum (lower) values. The dots correspond to individual values. Blue dots are those subjects without the comorbidity and the red ones are those who have it. Significant differences are indicated with a horizontal line and three asterisks (***) between the boxes. Abbreviations: Aβ, amyloid beta. P-tau, phosphorylated tau. Figure 1A refers to the first line where plasma Aβ40 values are compared according to the different comorbidities. Figure 1B refers to the second line where Aβ42 levels are compared; Figure 1C corresponds to the comparison of Aβ42/Aβ40 ratio values in the third line; Figure 1D corresponds to the fourth line, where p-tau181 values are compared according to the different comorbidities.

Regarding mean Aβ42 levels, they showed no differences between subjects with and without DM (26.6 vs. 23.9 pg/mL; *p*-value = 0.051), with and without HT (24.1 vs. 24.4 pg/mL; *p*-value = 0.77), with and without DLP (24.2 vs. 24.1 pg/mL; *p*-value = 0.94), or with and without a history of TBI (24.2 vs. 23.8 pg/mL; *p*-value = 0.75) (Figure 1B).

The Aβ42/Aβ40 ratio was significantly lower among diabetic subjects (0.076 vs. 0.083; *p*-value = 0.025; Cohen’s d = 0.65), and this difference remained significant after adjusting for eGFR (to rule out the possible influence of diabetic nephropathy) (0.077 vs. 0.083; *p*-value = 0.027). When we stratified by gender, the differences remained in women (0.07 vs. 0.08; *p*-value = 0.04) but not in men (0.07 vs. 0.08; *p*-value = 0.08). The Aβ42/Aβ40 ratio was also lower in hypertensive subjects (0.080 vs. 0.084; *p*-value = 0.035; Cohen’s d = 0.39). When we divided the sample in genders, this difference lost significance in both women (0.080 vs. 0.085; *p*-value = 0.07) and men (0.08 vs. 0.079; *p*-value = 0.87). However, they were not significantly lower among subjects with DLP (0.081 vs. 0.084; *p*-value = 0.086) or a history of TBI (0.081 vs. 0.083; *p*-value = 0.59) (Figure 1C).

The only factor that was shown to be associated with higher mean p-tau181 levels was HT (1.32 vs. 1.10 pg/mL; *p*-value = 0.004; Cohen’s d = 0.53). To rule out that this finding was mediated by a possible nephropathy in hypertensive patients, we adjusted the results for eGFR, and they remained significant (1.25 vs. 1.18 pg/mL; *p*-value = 0.004). In this case, when we stratified by gender, the differences remained significant in males (1.45 vs. 1.13 pg/mL; *p*-value = 0.03) but not in women (1.17 vs. 1.09 pg/mL; *p*-value = 0.33). The mean plasma p-tau181 was not significantly higher in subjects with DM (1.28 vs. 1.18 pg/mL; *p*-value = 0.39), DLP (1.2 vs. 1.17 pg/mL; *p*-value = 0.59), or a history of TBI (1.27 vs. 1.18 pg/mL; *p*-value = 0.47) (Figure 1D).

### 2.3. Influence of Comorbidity Status on CSF Markers

There were no differences in CSF Aβ40 levels in subjects with and without DM (10,921.82 vs. 11,362.4 pg/mL; *p*-value = 0.74), with and without HT (10,957.87 vs. 10,984.04 pg/mL; *p*-value = 0.96), with and without DLP (10,760.67 vs. 11,103.5 pg/mL; *p*-value = 0.57), or with and without TBI (10,924.93 vs. 11,373.36 pg/mL; *p*-value = 0.75).

The CSF Aβ42 levels were similar in subjects with and without DM (809.9 vs. 834.16 pg/mL; *p*-value = 0.86), with and without HT (763.4 vs. 844.4 pg/mL; *p*-value = 0.22), with and without DLP (796.26 vs. 822.98 pg/mL; *p*-value = 0.67), or with and without TBI (802.62 vs. 904.36 pg/mL; *p*-value = 0.45).

The Aβ42/Aβ40 ratio was not different between diabetic and non-diabetic subjects (0.073 vs. 0.074; *p*-value = 0.86) but was different between subjects with and without HT (0.069 vs. 0.078, *p*-value = 0.03; Cohen’s d = 0.43). This difference was no longer significant when stratifying the subjects into males (0.06 vs. 0.07; *p*-value = 0.25) and females (0.072 vs. 0.078; *p*-value = 0.21). There were also no differences among subjects with and without DLP (0.074 vs. 0.075; *p*-value = 0.90) or with a previous history of TBI (0.08 vs. 0.074; *p*-value = 0.29).

As for the CSF p-tau181 levels, they were similar in subjects with and without DM (47.3 vs. 46.9 pg/mL; *p*-value = 0.96), with and without HT (43.9 vs. 52.46 pg/mL; *p*-value = 0.16), with and without DLP (47.7 vs. 46.6 pg/mL; *p*-value = 0.85), and with and without TBI (47.9 vs. 41.5 pg/mL; *p*-value = 0.46).

### 2.4. Influence of Biometric and Biochemical Variables on Plasma Markers

**Figure 2:**
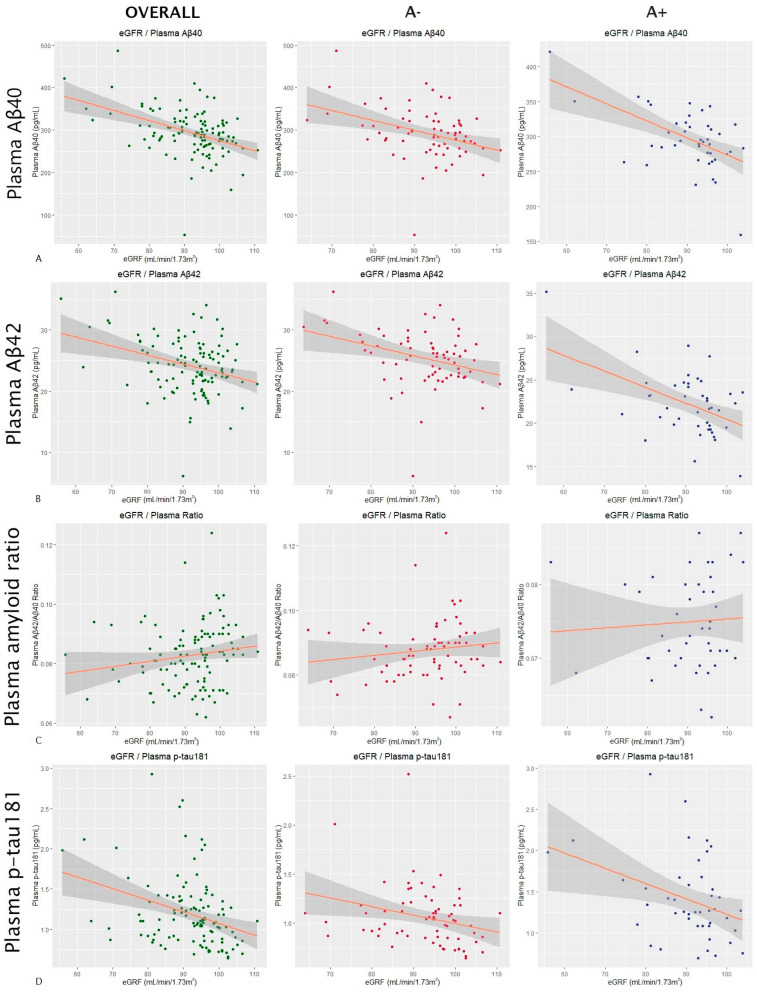
Influence of the glomerular filtration rate on AD plasma markers.

In the overall sample, eGFR was shown to have a significant effect (*p*-value < 0.0001) on the Aβ40 levels, with an estimated variation of −2.39 pg/mL per mL/min/1.73 m^2^ of filtrate. When stratified by amyloid groups, the effect of eGFR on the plasma Aβ40 levels was also significant both in A− subjects (Estimate = −2.35; *p*-value = 0.002) and in A+ subjects (Estimate = −2.6 pg/mL; *p*-value = 0.0006 in A+) (Figure 2A). The effect also remained significant after stratifying into females (Estimate = −1.85 pg/mL; *p*-value = 0.0002) and males (Estimate = −3.02 pg/mL; *p*-value = 0.009). AST also showed a significant influence on the plasma Aβ40 levels in the overall sample (Estimate = −2.67 pg/mL; *p*-value = 0.004) and in the A− subjects (Estimate = −4.2 pg/mL; *p*-value = 0.005). However, it was not significant in the A+ group (Estimate = −1.23 pg/mL; *p*-value = 0.25). In this case, AST showed an effect on the plasma Aβ40 levels in women (Estimate = −1.92 pg/mL; *p*-value = 0.03) and marginally in men (Estimate = −4.29 pg/mL; *p*-value = 0.0507). A similar trend was observed with the HDLc values. They showed a significant effect on Aβ40 both in the overall sample (Estimate = −0.94 pg/mL; *p*-value = 0.008) and in the A− subjects (Estimate = −1.12 pg/mL; *p*-value = 0.03), but this did not occur in the A+ group (Estimate = −0.65 pg/mL; *p*-value = 0.16). After adjusting the results for the influence of HDLc on plasma Aβ40 according to the *ApoE4* status, the results remained significant in the overall sample (Estimate = −0.93 pg/mL; *p*-value = 0.009) and in the A− subjects (Estimate = −1.13 pg/mL; *p*-value = 0.03). When stratifying by gender, in females, the effect of HDL on the plasma Aβ40 levels was significant (Estimate = −0.70 pg/mL; *p*-value = 0.04), and in males, it was close to statistical significance (Estimate = −1.87 pg/mL; *p*-value = 0.051).

The scatter plots in Figure 2 show the results of a multiple linear regression in which the influence of the estimated glomerular filtration rate on plasma levels of Aβ40 (row Figure 2A) and Aβ42 (row Figure 2B), the plasma Aβ42/Aβ40 ratio (row Figure 2C), and plasma levels of p-tau181 (row Figure 2D) was analyzed. All results were adjusted for age and sex. The first column shows the results for the overall sample, the second one for amyloid-negative subjects, and the third one for amyloid-positive subjects (according to CSF). The dots correspond to the individual values of each subject (green for the overall sample, red for A− subjects, and blue for A+). The orange line is the regression line, and the gray shaded area shows the confidence interval. Abbreviations: eGFR, estimated glomerular filtration rate. Aβ, amyloid beta. P-tau, phosphorylated tau. A, amyloid.

The eGFR also showed a significant effect on Aβ42 levels in the overall sample (Estimate = −0.21 pg/mL; *p*-value < 0.0001). In the A− group, the estimated variation was −0.17 pg/mL (*p*-value = 0.009), and in the A+ group, it was −0.22 pg/mL (*p*-value = 0.0005) (Figure 2B). After stratifying by gender, the effect remained significant in both females (Estimate = −0.18 pg/mL; *p*-value = 0.001) and males (Estimate = −0.23 pg/mL; *p*-value = 0.01). The other studied factors that were shown to have a marginal effect on Aβ42 levels were the total cholesterol, with an estimate for the global sample of −0.02 pg/mL (*p*-value = 0.04), an estimate of 0.02 pg/mL for A− subjects (*p*-value = 0.09), and an estimate of 0.0009 pg/mL for A+ subjects (*p*-value = 0.96), and HDLc, with an estimate for the global sample of −0.07 pg/mL (*p*-value = 0.02), an estimate of −0.07 pg/mL in the A− group (*p*-value = 0.08), and an estimate of −0.05 pg/mL in the A+ subjects (*p*-value = 0.18). The results of the influence of total cholesterol on plasma Aβ42 levels remained significant after adjusting for *ApoE4* status in the overall sample (Estimate = −0.025 pg/mL; *p*-value = 0.032). The same happened with HDLc (Estimate = −0.066 pg/mL; *p*-value = 0.026). In the case of total cholesterol, the effect disappeared after stratifying into females (Estimate = −0.01 pg/mL; *p*-value = 0.25) and males (Estimate = −0.05 pg/mL; *p*-value = 0.08). The effect of HDLc was not significant in females (Estimate = −0.04 pg/mL; *p*-value = 0.19), but it remained significant in males (Estimate = −0.19 pg/mL; *p*-value = 0.01).

For the plasma Aβ42/Aβ40 ratio, the eGFR showed no significant effects in either the overall sample (Estimate < 0.0001; *p*-value = 0.83) or the A− (Estimate < 0.0001; *p*-value = 0.5) or A+ (Estimate < 0.0001; *p*-value = 0.74) group (Figure 2C). Apart from the eGFR, only ALP showed a significant effect on the amyloid ratio in the overall sample (Estimate = 0.0001; *p*-value = 0.005), but not in the A+ (Estimate < 0.0001; *p*-value = 0.75) or A− group (Estimate = 0.0001; *p*-value = 0.5). The effect also did not remain after stratifying into women (Estimate < 0.0001; *p*-value = 0.99) and men (Estimate = 0.0004; *p*-value = 0.09).

For the plasma p-tau181 levels, the eGFR did not show a significant effect either in the overall sample (Estimate = −0.004 pg/mL; *p*-value = 0.39) or in the A− (Estimate = −0.003 pg/mL; *p*-value = 0.50) or A+ group (Estimate = −0.07 pg/mL; *p*-value = 0.38) (Figure 2D). For the plasma p-tau181 values, we also stratified by AD group, but we found no significant effects of the eGFR in either the AD− (Estimate = −0.006 pg/mL; *p*-value = 0.08) or the AD+ group (Estimate = −0.004 pg/mL; *p*-value = 0.8).

The effects of the remaining biochemical and biometric parameters on plasma markers are described in Table 2. The results when stratified by amyloid group can be found in Supplementary Table S1.

### 2.5. Influence of Biometric and Biochemical Variables on CSF Markers

In the overall sample, eGFR showed a significant influence on CSF Aβ40 levels (Estimate = 73.71 pg/mL; *p*-value = 0.04). However, when stratifying by amyloid group, statistical significance did not remain in either A− (Estimate = 83.87 pg/mL; *p*-value = 0.075) or A+ subjects (Estimate = 103.01 pg/mL; *p*-value = 0.071). This effect was maintained in males (Estimate = 106.1 pg/mL; *p*-value = 0.042) but not in females (Estimate = 43.1 pg/mL; *p*-value = 0.38). BMI also showed a significant relationship globally (Estimate = −150.3 pg/mL; *p*-value = 0.03) but not when stratifying by A− (Estimate = −132.18 pg/mL; *p*-value = 0.11) or A+ (Estimate = −174.9 pg/mL; *p*-value = 0.15) subjects. In this case, the effect remained in women (Estimate = −220.2 pg/mL; *p*-value = 0.004) but not in men (Estimate = 70.6 pg/mL; *p*-value = 0.64).

The CSF levels of Aβ42 were influenced in the overall sample by plasmatic total cholesterol (Estimate = −2.38 pg/mL; *p*-value = 0.009). When stratifying by amyloid group, this significance remained in both A− (Estimate = −2.71 pg/mL; *p*-value = 0.003) and A+ subjects (Estimate = 3.49 pg/mL; *p*-value = 0.01). These findings remained significant after adjusting for *ApoE4* status. In the overall sample, the estimate was −2.4 pg/mL (*p*-value = 0.006), in A− subjects, it was −2.7 pg/mL (*p*-value = 0.034), and in A+ subjects, it was 3.04 pg/mL (*p*-value = 0.03). In this case, when we stratified by gender, the effect remained significant in women (Estimate = −2.36 pg/mL; *p*-value = 0.024) but not in men (Estimate = −43 pg/mL; *p*-value = 0.19). The LDLc levels also showed an influence on the CSF levels of Aβ42 in the overall sample (Estimate = −2.79 pg/mL; *p*-value = 0.009) and in both A− (Estimate = −3.36 pg/mL; *p*-value = 0.002) and A+ subjects (Estimate = 4.09 pg/mg; *p*-value = 0.007). After adjusting for *ApoE4* status, the influence of LDLc on CSF Aβ42 levels remained significant in the overall sample (Estimate = −2.78 pg/mL; *p*-value = 0.007) and in A− (Estimate = −3.36 pg/mL; *p*-value = 0.002) and A+ subjects (Estimate = 3.68 pg/mL; *p*-value = 0.017). In the female group, the effect of LDLc on CSF Aβ42 remained significant (Estimate = −3.05 pg/mL; *p*-value = 0.01), but this was not the case for the male group (Estimate = −2.15 pg/mL; *p*-value = 0.35). The influence of ALP was significant in the overall sample (Estimate = 4.59 pg/mL; *p*-value = 0.01) and in the A+ subjects (Estimate = 6.86 pg/mL; *p*-value = 0.002) but not in the A− group (Estimate = 0.25 pg/mL; *p*-value = 0.98). This effect was only present in the male group (Estimate = 22.7 pg/mL; *p*-value = 0.01), but not in the female sample (Estimate = −3.76 pg/mL; *p*-value = 0.34).

The CSF Aβ42/Aβ40 ratio showed a significant relationship with plasma GGT in the overall sample (Estimate = −0.0002; *p*-value = 0.027) but not when stratifying into A− (Estimate = −0.00005; *p*-value = 0.45) or A+ groups (Estimate = −0.0001; *p*-value = 0.15). The effect of GGT was not present in women (Estimate = −0.0001; *p*-value = 0.2) or in men (Estimate = −0.0003; *p*-value = 0.11). ALP also showed a relationship with amyloid ratio in the overall sample (Estimate = 0.0004; *p*-value = 0.0002) and in the A+ group (Estimate = 0.0005; *p*-value = 0.0006) but not in the A− subjects (Estimate = 0.00004; *p*-value = 0.38). In this case, the effect remained significant in both women (Estimate = 0.0002; *p*-value = 0.01) and men (Estimate = 0.0006; *p*-value = 0.004).

Finally, the CSF levels of p-tau181 were shown to be influenced by ALP in the overall sample (Estimate = −0.36 pg/mL; *p*-value = 0.009) and in A+ subjects (Estimate = −0.78 pg/mL; *p*-value = 0.02) but not in the A− group (Estimate = 0.02 pg/mL; *p*-value = 0.77). After stratifying by gender, the influence was not present in males (Estimate = −0.61 pg/mL; *p*-value = 0.07) and was only marginally present in females (Estimate = −0.28 pg/mL; *p*-value = 0.049).

The effects of the remaining biochemical and biometric parameters on CSF markers are described in Table 3. These results when stratified by amyloid group can be found in Supplementary Table S2.

## 3. Discussion

For AD plasma markers to be useful in a clinical setting, it is essential to know the factors influencing their levels. In our cross-sectional research in CU subjects, we found that eGFR was the factor that was most consistently associated with changes in the plasma levels of Aβ40 and Aβ42.

As previously reported [7,10,11], we observed that the plasma levels of Aβ40 and Aβ42 had an inverse relationship with the glomerular filtration rate, and this relationship was significant in both A− and A+ subjects. This supports the hypothesis that the kidney plays an important role in plasma amyloid clearance [12]. In contrast, the eGFR did not show an effect on the amyloid ratio, which was likely because Aβ40 and Aβ42 were cleared in similar proportions. Although previous studies including subjects with cognitive impairment and dementia found an effect of filtration on the amyloid ratio, it was much weaker than on Aβ40 and Aβ42 separately [11]. This suggests that the amyloid ratio is a more stable marker than Aβ42 alone for use in the general population.

Unlike other studies that found a significant effect of the eGFR on plasma t-tau [7,10,11], we did not detect this effect on p-tau181 in either T+ or T− subjects. Most of these studies were carried out with sample sizes larger than ours, so this difference may be due to a lack of statistical power.

The other factor that showed an inverse effect on the Aβ40 and Aβ42 values was HDLc. This could be explained by the fact that HDLc has a protective cardiovascular effect, and subjects with higher HDLc levels may have had fewer vascular lesions that disrupted the blood–brain barrier. Also, a recent genetic association study pointed to elevated HDLc levels as a risk factor for AD, which could be related to a lower clearance of cerebral amyloid due to a lesser vascular load [13]. In this sense, we did not find any relationships with *ApoE4* status when considered as a potential confounding factor. However, although significant, the effects were marginal, and we found no differences in the plasma levels of Aβ40 and Aβ42 between healthy subjects and those previously diagnosed with dyslipidemia, even though the latter represented more than 60% of our population.

The opposite happened with diabetic and hypertensive subjects. Those with a previous diagnosis had lower Aβ42/Aβ40 ratios and, in the case of hypertensives, higher levels of p-tau181. However, this was not the case with the CSF markers. The analysis of punctual blood pressure and glycemia as continuous variables did not show an effect on the plasma biomarker levels. This was probably because the single values of each risk factor did not really have a significant effect, but the chronic damage that they produced in the blood–brain barrier (increasing its permeability) or on renal function (decreasing its clearance) did.

Plasma Aβ40 was shown to be increased in subjects with DM, but we did not find this pattern in CSF. In previous studies on this same cohort [14], we saw that the correlation between them was very weak, so this influence of DM may have been due both to a disruption in the blood–brain barrier and to a peripheral factor such as chronic renal damage. However, the reason for why diabetes did not affect plasma Aβ42 equally was not clear.

On the other hand, when we stratified these results by gender, in most cases, the differences ceased to be significant or were maintained only in the female group, which, in our sample, was the one with the largest sample size. This was possibly due to a lack of statistical power, especially in the male group.

Although our findings are generally consistent with what has been described so far, there are still issues to be resolved. For example, recent studies obtained contradictory conclusions regarding the effects of race on plasma markers. While some argued that it influenced their levels [15,16], others suggested that these differences may be due to disparities in analytical techniques and medical conditions [17]. Another aspect to consider in future studies is the influence that different drugs may have on plasma biomarker levels. In this regard, a recent article reported that subjects chronically treated with sacubitril/valsartan had lower levels of the AB42/AB40 ratio in plasma, which could lead to false positives in subjects with suspected AD [18].

Our research has several limitations. The most noteworthy are the small sample size and the lack of ethnic heterogeneity, which make it difficult to generalize our findings. In addition, this was cross-sectional research conducted in CU subjects, so longitudinal studies of patients at different stages of AD would be needed to extend our results. It should also be pointed out that, although we found an effect of the glomerular filtration rate on plasma Aβ in a continuous way, we did not carry out a group study that included controls and subjects with chronic kidney disease due to the small proportion of nephropathy in our sample.

## 4. Material and Methods

### 4.1. Participants

**Figure 3:**
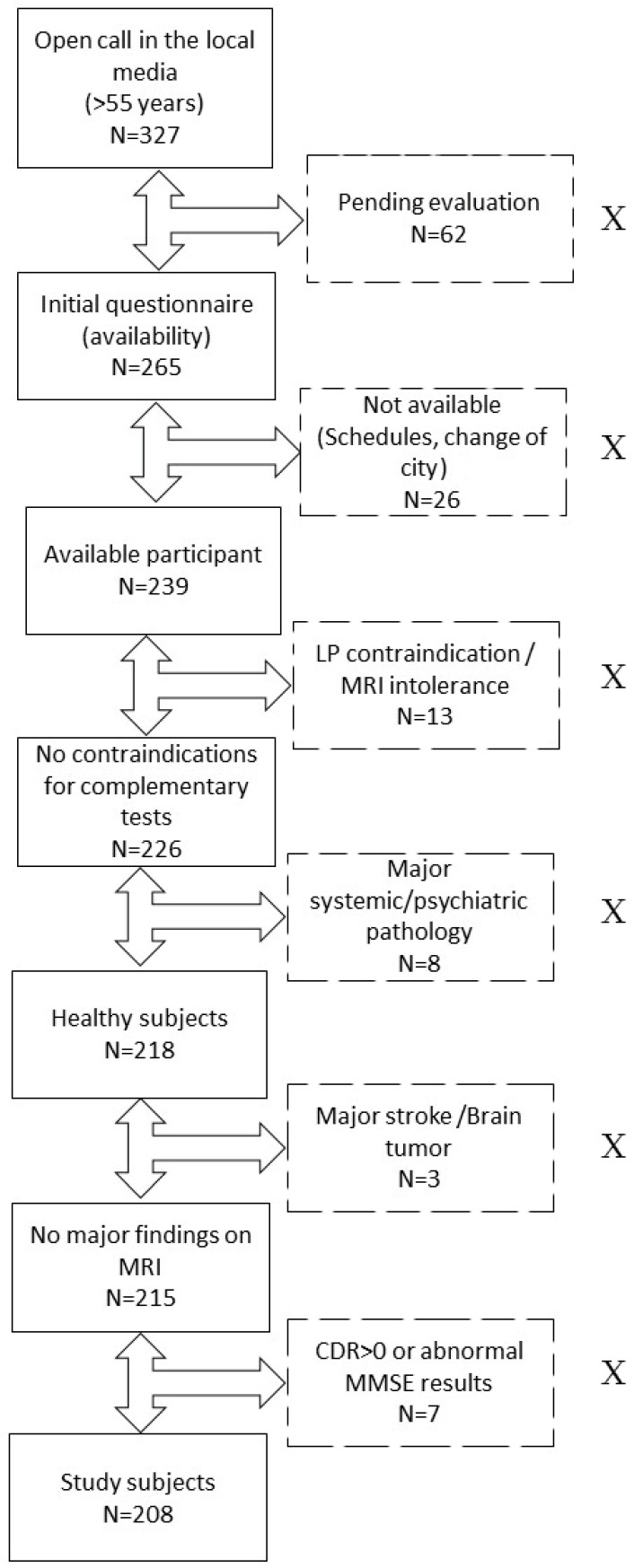
Recruitment process. Flowchart showing the process of recruitment and exclusion of study participants. Excluded participants are represented in boxes with dotted lines and marked with an “X”. Abbreviations: LP, lumbar puncture. MRI, magnetic resonance imaging. N, number of subjects. CDR, Clinical Dementia Eating. MMSE, Mini-Mental State Examination.

For this research, two hundred and eight subjects were evaluated. The study was approved by the ethics committee of the Marqués de Valdecilla University Hospital in Santander, Spain (Internal code: 2018.111). All participants belonged to the ‘Valdecilla Cohort for the study of memory and brain aging’ from our Cognitive Impairment Unit. This was a prospective cohort intended to longitudinally assess the preclinical stages of AD, and it was entirely composed of Caucasian CU volunteers [19]. The inclusion criteria were the following: (1) age ≥ 55 years; (2) consent for the extraction and storage of samples. The exclusion criteria were the following: (1) cognitive impairment determined by a Clinical Dementia Rating (CDR) [20] score > 0; (2) major systemic or psychiatric disease reviewed in their medical history; (3) any contraindications for performing the complementary tests (e.g., claustrophobia or anticoagulation). The detailed selection process can be found in previous work [20] and in Figure 3.

An in-depth initial questionnaire was used to collect demographic variables, such as the educational level, and to assess basic biometric measurements, including height, weight, and both systolic and diastolic blood pressure. All subjects underwent LP and blood extraction for the measurement of Aβ42, Aβ40, p-tau181, and total tau (t-tau) levels, along with biochemical, microbiological, and immunological determinations.

The project was approved by the ethics committee of the Hospital Universitario Marqués de Valdecilla, and all subjects signed an informed consent form.

### 4.2. Cognitive Evaluation

All subjects underwent a comprehensive neuropsychological assessment performed by neuropsychologists specialized in cognitive impairment. Relevant to this research, the Mini-Mental State Examination (MMSE) [21] was used for a global cognitive assessment, and the CDR score was used to establish the degree of dementia based on both cognition and functionality.

### 4.3. ApoE Status

Since the apolipoprotein E ε4 allele (*ApoE*-ε4) is the main genetic risk factor for late-onset AD [2], we studied the *ApoE* genotype in our participants. It was established using the TaqMan single-nucleotide polymorphism genotyping assay (Applied Biosystems, Foster City, CA, USA). Subjects carrying ≥ 1 copy of the ε4 allele were considered ε4+, and the rest were considered ε4−.

### 4.4. Sample Pre-Analysis

Both plasma and CSF samples were drawn on the same day (between 9 and 10 a.m.), with a time difference of less than 30 min with fasting subjects. Our center is part of the Alzheimer’s Association Quality Control program, so we complied with the standard recommendations for CSF collection and storage [22,23]. The lumbar punctures were performed between spaces L3 and L5, in the lateral decubitus, and with a standard 22-G needle. CSF was collected in 15 mL polypropylene tubes and centrifuged at room temperature for 10 min at 2000× *g*. The resultant was aliquoted in 500 µL volumes into 1 mL tubes and frozen at  −80 °C until analysis in the immunology laboratory of our hospital.

Plasma samples were also obtained by following the standardized procedure described previously [24]. The samples were collected in 10 mL EDTA tubes and kept cold until processing in the next 3 h. The samples were centrifuged at 1800× *g* for 10 min. The supernatant then was stored in volumes of 500 µL in polypropylene tubes and frozen at  −80 °C until analysis in the biochemistry laboratory of our hospital.

### 4.5. Biomarker Analysis

CSF Aβ40, Aβ42, p-tau181, and t-tau levels were measured with the automated Lumipulse G600 II immunoassay analyzer [19] (Fujirebio Diagnostics, Malvern, PA, USA) with the *Lumipulse G β-Amyloid 1–40* (lot 4YX3085), *Lumipulse G β-Amyloid 1–42* (lot 7ZX3084), *Lumipulse G p-tau181* (lot 5DX3055), and *Lumipulse G t-tau* (lot 6BX3064) kits (Fujirebio Diagnostics, Malvern, PA, USA). The lower limit of detection (LLD) for Aβ40 was 2.78 pg/mL. The intra- and inter-assay variation was <4.5 and <7.1%, respectively. The sensitivity for Aβ42 was 150 pg/mL, and the intra- and inter-assay variation was <4 and <5.9%, respectively. For t-tau and p-tau181, the LLDs were 80 pg/mL and 8 pg/mL, respectively. The intra-assay variation was <1.2 and <1.5%, and the inter-assay variation was <1.3 and 2.6%, respectively [25,26].

An unbiased Gaussian mixture modeling based on our population [27] was used to establish the cut-off points, and we classified subjects according to the ATN classification [28] while considering these cut-off points. We dichotomized the variables and considered a subject as Aβ-positive (A+) when the CSF Aβ42/Aβ40 ratio was <0.076, as tau-positive (T+) when p-tau181 > 73.2 pg/mL, and as neurodegeneration-positive (N+) when t-tau > 543 pg/mL. We also divided our sample into subjects with biologically defined Alzheimer’s pathology (A+ plus T+), referring to them as AD+ and the rest as AD−.

Fujirebio’s Lumipulse G600II was also used to measure plasma Aβ40, Aβ42, and p-tau181 values with the following kits: *Lumipulse G β-Amyloid 1–40 Plasma* (lot T4B3033), *Lumipulse G β-Amyloid 1–42 Plasma* (lot T6B3074), and *Lumipulse G pTau 181 Plasma* (lot T9B3084) (Fujirebio Diagnostics, Malvern, PA, USA). The analytical sensitivity for Aβ40, Aβ42, and p-tau181 was 0.44, 0.37, and 0.052 pg/mL, respectively. The intra-assay variation was <3.1, <3.8, and <2.3%, and the inter-run variability was <3.6, <4.7, and <3.9%, respectively.

### 4.6. Comorbidity Assessment

We considered the following continuous variables in serum: total cholesterol (TC) (mg/dL), low-density lipoprotein cholesterol (LDLc) (mg/dL), high-density lipoprotein cholesterol (HDLc) (mg/dL), albumin (g/dL), total bilirubin (mg/dL), gamma-glutamyl transferase (GGT), alanine aminotransferase (ALT) (U/L), aspartate aminotransferase (AST) (U/L), alkaline phosphatase (ALP) (U/L), glucose (mg/dL), and estimated glomerular filtration rate (eGFR) obtained through the Chronic Kidney Disease Epidemiology Collaboration (CKD-EPI) formula [29] (mL/min/1.73 m^2^). The body mass index (BMI) (kg/m^2^) was calculated with the height (m) and weight (kg) data collected in the initial assessment. Systolic and diastolic blood pressure (SBP and DBP) was measured in mmHg. Subjects were also dichotomized according to their medical records into those who had a previous history of hypertension (HT), DM, DLP, or traumatic brain injury (TBI) and those who had not.

### 4.7. Statistical Analysis

The Shapiro–Wilk test was used to assess the distribution of the variables. For descriptive analysis, the mean and standard deviation (SD) or median and interquartile range (IQR) were used as appropriate. A log10-transformation of all continuous variables was performed to meet the assumption of normality.

ANCOVA was used to assess differences in both plasma and CSF levels of Aβ40, Aβ42, and p-tau181 as a function of the dichotomous variables HT, DLP, DM, and TBI, considering both age and sex. Subsequently, a post hoc Sidak test was performed to evaluate the differences between groups. We used Cohen’s d to study the effect size in significant results.

The influence of the continuous physiological variables on AD plasma and CSF markers was assessed using a multiple linear regression model adjusted by sex and age both in the overall sample and when stratifying into the A+/− groups. Since the *ApoE* gene is involved in lipid metabolism, the results with different cholesterol values were adjusted according to the *ApoE4* status to rule this out as a potential confounding factor.

All analyses were performed with R version 4.2.2 (R Foundation for Statistical Computing, Vienna, Austria). A *p*-value of <0.05 was taken as statistical significance.

## 5. Conclusions

In conclusion, renal function is the physiological factor that most influences plasma Aβ40 and Aβ42 levels in CU subjects. However, the Aβ42/Aβ40 ratio does not seem to be altered for this factor, so it could be a more stable marker as a screening tool in the general population.

## Figures and Tables

**Table 1:** Sample description.

Characteristic	Overall n = 208	Females n = 136	Males n = 72
Females/Males (%)		65.4%	34.6%
Age, median (IQR)	64 (60–69)	64 (59–68)	68 (62–73)
*ApoE* ε4 carrier, n. (%)	63 (30.3%)	42 (30.9%)	21 (29.2%)
MMSE (0–30), median (IQR)	29 (28–30)	29 (28–30)	29 (28–30)
Education (years), median (IQR)	12 (12–18)	12 (12–18)	12 (8–18)
**Comorbidities**			
Hypertension (% yes)	39.1	27.3	64.9
Diabetes (% yes)	10.4	11.7	8.1
Dyslipidemia (% yes)	60.9	59.7	62.2
Traumatic Brain Injury (% yes)	8.2	9.1	10.8
Body Mass Index, median (IQR), {reference values}, kg/m^2^	26.3 (23.6–29.5) {18.5–24.9}	25.8 (23.2–28.7)	28.1 (25.3–30.3)
Systolic Blood Pressure, mean (SD), {reference values}, mmHg	144.2 (15.1) {120–139}	141.8 (15.8)	148.7 (12.2)
Diastolic Blood Pressure, mean (SD), {reference values}, mmHg	84.4 (10.1) {80–90}	83.0 (9.5)	86.9 (10.6)
eGFR, median (IQR), {reference values}, mL/min/1.73 m^2^	94.5 (87.3–97.9) {>60}	94.8 (89.4–99.1)	90.6 (82.9–95.3)
Glucose, median (IQR), {reference values}, mg/dL	93 (86–100) {74–109}	93 (86–97)	93 (87–105)
Total cholesterol, mean (SD), {reference values}, mg/dL	197.3 (36.9) {150–240}	206.8 (34.4)	177.5 (34.8)
LDL cholesterol, mean (SD), {reference values}, mg/dL	118.6 (30.3) {100–129}	123.8 (29.9)	107.8 (28.7)
HDL cholesterol, mean (SD), {reference values}, mg/dL	58.2 (15.4) {40–59}	63.1 (14.6)	48.1 (12.0)
AST, median (IQR), {reference values}, U/L	22 (20–25) {14–35}	22 (19–25)	23 (21–26)
ALT, median (IQR), {reference values}, U/L	20 (16–25) {10–49}	19 (16–23)	23 (18–27)
GGT, median (IQR), {reference values}, U/L	20 (13–30) {6–73}	16 (13–24)	27 (21–37)
ALP, mean (SD), {reference values}, U/L	67.8 (17.7) {46–116}	70.2 (18.3)	63.0 (15.8)
Total Bilirubin, median (IQR), {reference values}, mg/dL	0.6 (0.4–0.7) {0.2–1.1}	0.5 (0.4–0.6)	0.6 (0.5–0.8)
Plasma Albumin, mean (SD), {reference values}, g/dL	4.3 (0.2) {3.8–5.1}	4.3 (0.2)	4.2 (0.2)
**Plasma Biomarkers**			
Aβ40, median (IQR), pg/mL	292.8 (266–318)	293.5 (268.8–319.8)	282.9 (262.7–327.0)
Aβ42, median (IQR), pg/mL	23.6 (21.5–26.3)	24.7 (22.2–27.0)	22.8 (20.1–26.7)
Ratio Aβ42/40, median (IQR)	0.082 (0.074–0.089)	0.084 (0.079–0.09)	0.079 (0.071–0.084)
P-tau181, median (IQR), pg/mL	1.1 (0.9–1.4)	1.1 (0.9–1.3)	1.2 (1.0–1.5)
**CSF Biomarkers**			
Aβ40, mean (SD), pg/mL	10,850.7 (3191.4)	10,640.7 (3256.7)	11,649.6 (3096.3)
Aβ42, median (IQR), pg/mL	819.5 (577–1037)	761 (565–1013)	782 (501–1157)
Ratio Aβ42/40, median (IQR)	0.084 (0.065–0.093)	0.082 (0.069–0.090)	0.078 (0.048–0.088)
P-tau181, median (IQR), pg/mL	37.5 (30.4–54.3)	35.5 (27–47.2)	51.6 (35.8–61.6)
**ATN group, n. (%)**			
A−T−N−	135 (64.9%)	91 (66.9%)	44 (61.1%)
A+T−N−	50 (24%)	34 (25%)	16 (22.2%)
A−T+N−	1 (0.5%)	1 (0.7%)	0
A+T+Nx	22 (10.6%)	10 (7.3%)	12 (16.7)

Overall sample description. Abbreviations: n, number of subjects. IQR, interquartile range. MMSE, Mini-Mental State Examination. CSF, cerebrospinal fluid. Aβ, amyloid beta. SD, standard deviation. LDL, low-density lipoprotein. HDL, high-density lipoprotein. AST, aspartate aminotransferase. ALT, alanine aminotransferase. GGT, gamma-glutamyl transferase. ALP, alkaline phosphatase. eGFR, estimated glomerular filtration rate. P-tau, phosphorylated tau. A, amyloid. T, tau. N, neurodegeneration. Nx, both positive and negative neurodegeneration groups.

**Table 2:** Influences of physiological variables on plasma markers in the overall sample.

		Aβ40 (pg/mL)	Aβ42 (pg/mL)	Aβ42/Aβ40	p-tau181 (pg/mL)
**eGFR (mL/min/1.73 m^2^)**	**Estimate**	−2.39	−0.21	0.00001	−0.037
	***p*** **-value**	**<0.0001**	**<0.0001**	0.83	0.39
**BMI (kg/m^2^)**	**Estimate**	0.39	−0.05	−0.0002	0.01
	***p*** **-value**	0.74	0.60	0.23	0.22
**Glucose** **(mg/dL)**	**Estimate**	0.23	0.02	0.00001	−0.001
	***p*** **-value**	0.25	0.19	0.99	0.45
**GGT** **(U/L)**	**Estimate**	−0.32	−0.04	0.00006	−0.0002
	***p*** **-value**	0.25	0.08	0.18	0.89
**AST** **(U/L)**	**Estimate**	−2.67	−0.15	0.0003	0.003
	***p*** **-value**	**0.004**	0.059	0.09	0.60
**ALT** **(U/L)**	**Estimate**	−0.87	−0.05	0.00006	−0.003
	***p*** **-value**	0.14	0.31	0.57	0.47
**ALP** **(U/L)**	**Estimate**	−0.22	0.02	0.0001	−0.002
	***p*** **-value**	0.44	0.32	**0.005**	0.33
**Total Bilirubin (mg/dL)**	**Estimate**	−12.07	−0.73	0.0001	−0.05
	***p*** **-value**	0.49	0.62	0.97	0.65
**Albumin** **(g/dL)**	**Estimate**	22.15	0.54	−0.004	0.07
	***p*** **-value**	0.35	0.79	0.28	0.66
**Total Cholesterol** **(mg/dL)**	**Estimate**	−0.27	−0.02	−0.00001	−0.0006
	***p*** **-value**	0.06	**0.04**	0.46	0.56
**LDLc (mg/dL)**	**Estimate**	−0.20	−0.02	−0.00003	−0.0003
	***p*** **-value**	0.23	0.12	0.31	0.76
**HDLc (mg/dL)**	**Estimate**	−0.94	−0.07	0.00003	−0.001
	***p*** **-value**	**0.008**	**0.02**	0.68	0.63
**SBP (mmHg)**	**Estimate**	−0.38	−0.03	0.000002	0.001
	***p*** **-value**	0.27	0.20	0.97	0.64
**DBP (mmHg)**	**Estimate**	−0.28	−0.04	−0.00003	−0.0008
	***p*** **-value**	0.59	0.30	0.71	0.83

Table 2 shows the results of multiple linear regressions in which plasma markers were selected as the dependent variable and the different biochemical and biometric values were individually selected as independent variables while adjusting for age and sex. The Estimate represents the number of units (pg/mL when applicable) by which the AD plasma markers varied for each unit by which the studied parameter was modified. The *p*-value is the value of statistical significance. Significant results are highlighted in bold. Abbreviations: eGFR, estimated glomerular filtration rate. BMI, body mass index. LDLc, low-density lipoprotein cholesterol. HDLc, high-density lipoprotein cholesterol. AST, aspartate aminotransferase. ALT, alanine aminotransferase. GGT, gamma-glutamyl transferase. ALP, alkaline phosphatase. SBP, systolic blood pressure. DBP, diastolic blood pressure. Aβ, amyloid beta. p-tau, phosphorylated tau.

**Table 3:** Influences of physiological variables on CSF markers in the overall sample.

		Aβ40 (pg/mL)	Aβ42 (pg/mL)	Aβ42/Aβ40	p-tau181 (pg/mL)
**eGFR (mL/min/1.73 m^2^)**	**Estimate**	73.71	3.87	−0.0001	0.47
	***p*** **-value**	**0.039**	0.32	0.60	0.12
**BMI (kg/m^2^)**	**Estimate**	−150.32	−9.42	0.0001	−0.76
	***p*** **-value**	**0.027**	0.20	0.73	0.19
**Glucose** **(mg/dL)**	**Estimate**	1.24	0.78	0.00005	−0.16
	***p*** **-value**	0.91	0.54	0.46	0.09
**GGT** **(U/L)**	**Estimate**	−0.24	−3.24	−0.0002	0.05
	***p*** **-value**	0.98	0.06	**0.027**	0.71
**AST** **(U/L)**	**Estimate**	−83.1	−4.79	0.0001	−0.63
	***p*** **-value**	0.13	0.42	0.63	0.18
**ALT** **(U/L)**	**Estimate**	4.27	1.37	0.00006	−0.36
	***p*** **-value**	0.90	0.71	0.78	0.22
**ALP** **(U/L)**	**Estimate**	1.77	4.59	0.0003	−0.37
	***p*** **-value**	0.91	**0.01**	**0.0002**	**0.009**
**Total Bilirubin (mg/dL)**	**Estimate**	−1459.05	−191.07	−0.004	6.08
	***p*** **-value**	0.15	0.08	0.51	0.48
**Albumin** **(g/dL)**	**Estimate**	712.7	−9.20	−0.006	−4.04
	***p*** **-value**	0.60	0.95	0.51	0.73
**Total Cholesterol** **(mg/dL)**	**Estimate**	−15.5	−2.38	−0.00009	0.038
	***p*** **-value**	0.07	**0.009**	0.09	0.59
**LDLc (mg/dL)**	**Estimate**	−15.08	−2.79	0.0001	0.05
	***p*** **-value**	0.13	**0.009**	0.056	0.55
**HDLc (mg/dL)**	**Estimate**	−15.7	−1.37	0.000006	0.18
	***p*** **-value**	0.46	0.55	0.96	0.30
**SBP (mmHg)**	**Estimate**	−28.59	−2.34	−0.000001	−0.08
	***p*** **-value**	0.15	0.28	0.99	0.67
**DBP (mmHg)**	**Estimate**	−17.04	−0.79	0.00008	−0.34
	***p*** **-value**	0.58	0.81	0.67	0.19

Table 3 shows the results of multiple linear regressions in which cerebrospinal fluid markers were selected as the dependent variable and the different biochemical and biometric values were individually selected as independent variables while adjusting for age and sex. The Estimate represents the number of units (pg/mL when applicable) by which the markers varied for each unit by which the studied parameter was modified. The *p*-value is the value of statistical significance. Significant results are highlighted in bold. Abbreviations: CSF, cerebrospinal fluid. eGFR, estimated glomerular filtration rate. BMI, body mass index. LDLc, low-density lipoprotein cholesterol. HDLc, high-density lipoprotein cholesterol. AST, aspartate aminotransferase. ALT, alanine aminotransferase. GGT, gamma-glutamyl transferase. ALP, alkaline phosphatase. SBP, systolic blood pressure. DBP, diastolic blood pressure. Aβ, amyloid beta. p-tau, phosphorylated tau.

## Data Availability

The data presented in this study are available on request from the corresponding author.

## Supplementary Materials

The following supporting information can be downloaded at: https://www.mdpi.com/article/10.3390/ijms25031481/s1.
