# Supplementary material for: Influence of Physiological Variables and Comorbidities on Plasma Aβ40, Aβ42, and p-tau181 Levels in Cognitively Unimpaired Individuals
Source: Int J Mol Sci. 2024 Jan 25;25(3):1481. doi: 10.3390/ijms25031481 (PMC10855058; doi:10.3390/ijms25031481)
Supplement: Supplementary file 1 [file ijms-25-01481-s001.zip › ijms-2815772-supplementary.pdf]

Supplementary file S1

Supplementary Table S1: Influence of physiological variables on plasma markers stratified by amyloid group.

		**Aβ40 (pg/ml)**		**Aβ42 (pg/ml)**		**Aβ42/Aβ40**		**p-tau181 (pg/ml)**	
		**A-**	**A+**	**A-**	**A+**	**A-**	**A+**	**A-**	**A+**
**eGFR (ml/min/1.73m²)**	**Estimate**	-2.33	-2.46	-0.18	-0.22	<0.0001	<0.0001	-0.003	-0.007
	**p-value**	**0.001**	**0.004**	**0.009**	**0.0005**	0.41	0.43	0.50	0.38
**BMI (kg/m2)**	**Estimate**	0.48	0.25	-0.09	-0.01	<0.0001	<0.0001	0.01	0.02
	**p-value**	0.77	0.88	0.45	0.94	0.08	0.73	0.16	0.31
**Glucose (mg/dl)**	**Estimate**	0.76	-0.19	0.06	-0.02	<0.0001	<0.0001	0.001	-0.001
	**p-value**	**0.02**	0.36	**0.02**	0.28	0.45	0.54	0.41	0.61
**GGT (U/l)**	**Estimate**	0.31	-0.54	0.02	-0.05	<0.0001	<-0.0001	-0.003	-0.0001
	**p-value**	0.60	**0.033**	0.64	**0.036**	0.60	0.67	0.24	0.97
**AST (U/l)**	**Estimate**	-4.2	-1.23	-0.23	-0.09	0.0005	<0.0001	0.001	0.005
	**p-value**	**0.004**	0.24	**0.04**	0.32	**0.02**	0.98	0.87	0.62
**ALT (U/l)**	**Estimate**	-1.11	-0.84	-0.05	-0.06	0.0001	<0.0001	-0.005	-0.003
	**p-value**	0.35	0.14	0.58	0.26	0.53	0.75	0.37	0.59
**ALP (U/l)**	**Estimate**	-0.62	0.55	-0.03	0.08	<0.0001	0.0001	0.003	-0.008
	**p-value**	0.12	0.19	0.29	**0.02**	0.35	**0.04**	0.11	0.055
**Total Bilirubin (mg/dl)**	**Estimate**	-12.1	-13.8	-0.42	-1.06	0.001	0.0001	-0.01	-0.30
	**p-value**	0.61	0.61	0.81	0.66	0.72	0.97	0.92	0.29
**Albumin (g/dl)**	**Estimate**	10.2	43.4	1.00	2.26	0.0001	-0.003	0.003	0.04
	**p-value**	0.77	0.13	0.71	0.37	0.97	0.39	0.98	0.88
**Total Cholesterol (mg/dl)**	**Estimate**	-0.33	-0.04	-0.02	0.007	<0.0001	<0.0001	-0.001	-0.0006
	**p-value**	0.08	0.87	0.09	0.74	0.89	0.39	0.21	0.82
**LDLc (mg/dl)**	**Estimate**	-0.29	0.09	-0.02	0.02	<0.0001	<0.0001	-0.001	-0.0004
	**p-value**	0.21	0.73	0.19	0.42	0.83	0.35	0.33	0.89
**HDLc (mg/dl)**	**Estimate**	-1.12	-0.65	-0.07	-0.06	<0.0001	<0.0001	-0.002	0.0005

	**p-value**	**0.03**	0.16	0.07	0.15	0.30	0.70	0.31	0.92
**SBP (mmHg)**	**Estimate**	-0.62	-0.17	-0.05	-0.02	<0.0001	<0.0001	0.003	-0.001
	**p-value**	0.28	0.63	0.23	0.60	0.65	0.94	0.29	0.75
**DBP /mmHg)**	**Estimate**	-0.41	-0.13	-0.07	-0.01	<0.0001	<0.0001	0.004	-0.005
	**p-value**	0.64	0.82	0.29	0.78	0.79	0.92	0.24	0.39

Supplementary table S1 shows the results of a multiple linear regressions in which plasma markers were selected as the dependent variable and the different biochemical and biometric values individually as independent variables, adjusting for age and sex. The table shows the results stratified by amyloid group according to cerebrospinal fluid values. A- are those subjects considered as amyloid negative and A+ are the amyloid positive ones. The Estimate represents the number of units (pg/ml when applicable) that the AD plasma markers vary for each unit that the studied parameter is modified. The p-value is the value of statistical significance, significant results are highlighted in bold. Abbreviations: eGFR, estimated glomerular filtration rate. BMI, body mass index. LDLc, low-density lipoprotein cholesterol. HDLc, high-density lipoprotein cholesterol. AST, aspartate aminotransferase. ALT, alanine aminotransferase. GGT, gamma-glutamyl transferase. ALP, alkaline phosphatase. SBP, systolic blood pressure. DBP, diastolic blood pressure. Aβ, amyloid beta. p-tau, phosphorylated tau.

Supplementary Table S2: Influence of physiological variables on CSF markers stratified by amyloid group.

		**Aβ40 (pg/ml)**		**Aβ42 (pg/ml)**		**Aβ42/Aβ40**		**p-tau181 (pg/ml)**	
		**A-**	**A+**	**A-**	**A+**	**A-**	**A+**	**A-**	**A+**
**eGFR (ml/min/1.73m²)**	**Estimate**	73.9	82.0	8.29	2.57	0.0001	-0.0001	0.10	0.84
	**p-value**	0.11	0.15	0.055	0.56	0.12	0.68	0.52	0.18
**BMI (kg/m2)**	**Estimate**	-132.2	-174.9	-13.25	-7.59	0.0003	-0.0002	-0.49	-0.04
	**p-value**	0.11	0.15	0.08	0.43	0.53	0.23	0.08	0.76
**Glucose (mg/dl)**	**Estimate**	-2.19	8.79	-0.55	0.41	<-0.0001	<-0.0001	-0.09	-0.07
	**p-value**	0.90	0.58	0.74	0.74	0.47	0.81	0.12	0.66
**GGT (U/l)**	**Estimate**	-73.6	29.7	-6.91	0.31	<0.0001	<-0.0001	-0.23	0.05
	**p-value**	**0.02**	0.11	**0.02**	0.83	0.35	0.23	**0.02**	0.81
**AST (U/l)**	**Estimate**	-119.5	-59.4	-9.10	-3.73	0.0001	<-0.0001	-0.24	-0.89
	**p-value**	0.13	0.45	0.21	0.53	0.42	0.95	0.37	0.29

**ALT (U/l)**	**Estimate**	16.2	-11.7	1.48	-0.11	<0.0001	<-0.0001	0.01	-0.51
	**p-value**	0.79	0.78	0.79	0.97	0.86	0.98	0.94	0.27
**ALP (U/l)**	**Estimate**	-6.31	15.8	0.02	6.86	<0.0001	0.0004	0.02	-0.78
	**p-value**	0.76	0.61	0.98	**0.002**	0.28	**0.0006**	0.77	**0.016**
**Total Bilirubin (mg/dl)**	**Estimate**	-2597.2	1309.1	-218.1	7.75	-0.001	-0.0009	-6.91	18.2
	**p-value**	**0.03**	0.51	0.055	0.96	0.69	0.92	0.09	0.40
**Albumin (g/dl)**	**Estimate**	236.7	1765.9	2.02	280.3	-0.002	0.01	-0.04	-26.5
	**p-value**	0.89	0.41	0.99	0.08	0.58	0.18	0.99	0.25
**Total Cholesterol (mg/dl)**	**Estimate**	-28.5	32.9	-2.71	3.49	<0.0001	<0.0001	-0.05	0.14
	**p-value**	**0.004**	0.06	**0.003**	**0.01**	0.28	0.35	0.11	0.48
**LDLc (mg/dl)**	**Estimate**	-34.3	47.15	-3.36	4.09	<-0.0001	<0.0001	-0.07	0.19
	**p-value**	**0.003**	**0.02**	**0.002**	**0.007**	0.13	0.47	0.058	0.37
**HDLc (mg/dl)**	**Estimate**	-17.9	-8.09	-0.83	-0.44	<0.0001	<-0.0001	0.04	0.30
	**p-value**	0.51	0.81	0.74	0.87	0.18	0.95	0.67	0.42
**SBP (mmHg)**	**Estimate**	-50.8	-10.9	-3.61	-0.28	<0.0001	<0.0001	-0.08	-0.20
	**p-value**	0.08	0.69	0.19	0.89	0.51	0.74	0.39	0.49
**DBP /mmHg)**	**Estimate**	-57.2	24.2	-3.55	2.59	0.0001	0.0001	-0.11	-0.43
	**p-value**	0.21	0.57	0.40	0.44	0.27	0.55	0.47	0.35

Supplementary table S2 shows the results of a multiple linear regressions in which cerebrospinal fluid markers were selected as the dependent variable and the different biochemical and biometric values individually as independent variables, adjusting for age and sex. The table shows the results stratified by amyloid group according to cerebrospinal fluid values. A- are those subjects considered as amyloid negative and A+ are the amyloid positive ones. The Estimate represents the number of units (pg/ml when applicable) that the AD plasma markers vary for each unit that the studied parameter is modified. The p-value is the value of statistical significance, significant results are highlighted in bold. Abbreviations: eGFR, estimated glomerular filtration rate. BMI, body mass index. LDLc, low-density lipoprotein cholesterol. HDLc, high-density lipoprotein cholesterol. AST, aspartate aminotransferase. ALT, alanine aminotransferase. GGT, gamma-glutamyl transferase. ALP, alkaline phosphatase. SBP, systolic blood pressure. DBP, diastolic blood pressure. Aβ, amyloid beta. p-tau, phosphorylated tau.
